# Bridging the gap: Leveraging telemedicine and IT infrastructure to connect outpatient oncology practices with specialized expert teams in the management of rare tumors

**DOI:** 10.1177/20552076241272709

**Published:** 2024-10-09

**Authors:** Julia Kasprzak, Timothy Goering, Karin Berger-Thürmel, Vanessa Kratzer, Wuthichai Prompinit, Sven P. Wichert, Simon Leutner, Norbert Langermann, Michael von Bergwelt-Baildon, Volker Heinemann, Hana Algül, Martin Zünkeler, Daniel Nasseh, on behalf of the TARGET Group

**Affiliations:** 1 Comprehensive Cancer Center Munich, LMU 27192 University Hospital, LMU Munich, Munich, Germany; 2 KAIROS GmbH - an IQVIA business, Bochum, Germany; 3 Department of Medicine III, LMU 27192 University Hospital, LMU Munich, Munich, Germany; 4 Comprehensive Cancer Center Munich, 27190 Klinikum Rechts der Isar, Technical University of Munich, Munich, Germany; 5 Department of Psychiatry and Psychotherapy, LMU University Hospital, LMU Munich, Munich, Germany; 6 Department of Medical Technology and IT, LMU University Hospital, LMU Munich, Munich, Germany; 7 553465 German Cancer Consortium (DKTK), Partner Site Munich, Munich, Germany; 8 Department of Internal Medicine II, Klinikum Rechts der Isar, Technical University of Munich, Munich, Germany

**Keywords:** Electronic health record, patient-reported outcome, telemedicine, outreach

## Abstract

**Objectives:**

The treatment of rare tumors often necessitates the involvement of highly specialized teams, typically based in larger medical centers or university hospitals, which are often lacking in rural areas. The German TARGET (the Trans-sectoral Personalized Care Concept for Patients with Rare Cancers) project aims to improve the network between outpatient oncology practices and more centralized expert teams via telemedicine.

**Methods:**

The primary work involved conceptualizing the implementation of project requirements based on feedback from various TARGET project teams, and ultimately, the method of implementation using the software CentraXX. Key requirements included the utilization of an electronic health record (EHR), incorporating appropriate access mediums such as smartphones, and utilizing user-specific certificates to ensure secure and tailored access. The implementation considered technical aspects, data protection regulations, and the need for user-friendly interfaces, particularly for older patients with cancer with limited technological proficiency.

**Results:**

The results detail the successful implementation of the project requirements using CentraXX, which facilitated the implementation of an EHR, access mediums (patient app), and browser access for outpatient doctors, addressing the project's technical, security, and usability needs.

**Conclusion:**

This article presents an overview of the requirements associated with the TARGET project and outlines how they were met in terms of the IT infrastructure. By focusing on the IT implementation rather than the medical trial results, this work aims to provide valuable insights and guidance for similar projects seeking to improve telemedicine networks and digital information exchange in the context of rare cancer treatment.

## Introduction

The treatment of rare tumors benefits from specialized centers, which are often lacking in rural regions.^1^ Telemedicine has the potential to bridge the gap between expert centers and local healthcare facilities.^2^ Consequently, the establishment of new and innovative telemedicine applications and IT networks among decentralized oncologists and specialized clinics remains a significant challenge. This issue is particularly critical in Germany, where digitalization efforts lag behind those of other European countries and the United States, as evidenced by its comparatively lower digitalization ranking.^3^

Implementing an EHR accessible to both oncologists in rural regions, specialist physicians, and patients can provide a potential networking solution between all relevant stakeholders. However, telemedical integration efforts must not only ensure technical functionality and privacy protection but also prioritize usability to avoid frustration.^4–6^

The TARGET project^7,8^ aims to address this challenge by involving primary care physicians in South Bavaria who recruit patients with rare cancers and optimizes communication with the Comprehensive Cancer Center Munich (CCCM)^9^ through a telemedicine-connected EHR system. Physicians remain consistently informed, and patients maintain access to their data throughout the process, all while considering data protection regulations. TARGET focuses on patients with rare tumors, and the concept will undergo comprehensive evaluation with the goal of improvement of care coordination.

To avoid confusion: this article aims to present the comprehensive IT concept and implementation of the TARGET project, highlighting efforts to enhance usability and minimize digital stress factors, and to serve as a model for other projects facing similar challenges and looking for architectural guidance. Therefore, the result of this article will be the sharing of knowledge about the IT concept itself. It does not focus on the quantitative and qualitative results of the actual trial, which is still ongoing until the end of 2025. The results of the trial and the final evaluation will be published in separate papers, which, unlike this work, will not focus as heavily on IT aspects.

## Methods

TARGET is a large-scale network project involving numerous teams from various disciplines, including, medical, evaluation, psycho-oncology, internal IT-coordination, external IT, and data center teams, all contributing to the project's development. Each team has formulated its specific requirements. It was then the responsibility of the internal IT-coordination team, consisting of two individuals, to translate these requirements into a technical concept and implement it with the help of others. Therefore, the major work to bring the IT architecture to life involved conceptualization, system configuration, and communication. The following explains the IT-related requirements, with bold text in brackets for ease of reference.

- (**EHR**) The central component of the TARGET project should represent the interplay between patients, primary care physicians, and clinicians of the CCCM. To achieve this, an EHR, similar to other currently (2023) ongoing German reference projects, was deemed appropriate.^10–12^
- (**Documentation**) The EHR must be capable of capturing the most important oncological parameters such as tumor diagnosis, histology, performed therapies, and other research-relevant information as defined by the project partners.
- (**Physician-access**) Physicians must be able to interact with the EHR from their practice computers, for example, uploading medical reports.
- (**Patient-access**) Patients should also be able to interact with the EHR via mobile devices. (**Authorization**) Since patients should not have full access to the EHR, a complex authorization concept must be implemented.
- (**One-framework**) All three components should be derived from one software framework. Alternatively, interfaces must be created for decoupled systems.
- (**Data-protection**) The central EHR must be hosted in the hospital and meet all data protection requirements.
- (**Synchronization**) The information flow between clinicians and primary care physicians should be as synchronized as possible. Therefore, information from the hospital's records should also be fed into the EHR.
- (**Usability-physicians**) Usability obstacles must be minimized and should not hinder the work of the recruiting centers. For recruiting centers, digital recruitment should not be significantly more complicated than paper-based recruitment.
- (**Usability-patients**) Patients, particularly the elderly, must be able to work with the technology effectively, with minimal barriers.
- (**Reporting**) The system should operate dynamically and inform physicians and patients about events. For example, if new surveys are available to a patient, they should be notified.
- (**Messaging**) The system should also support direct communication among all three stakeholders.
- (**Evaluation**) All data must be evaluable and usable by the expert teams by the end of the project.

**Figure 1.**
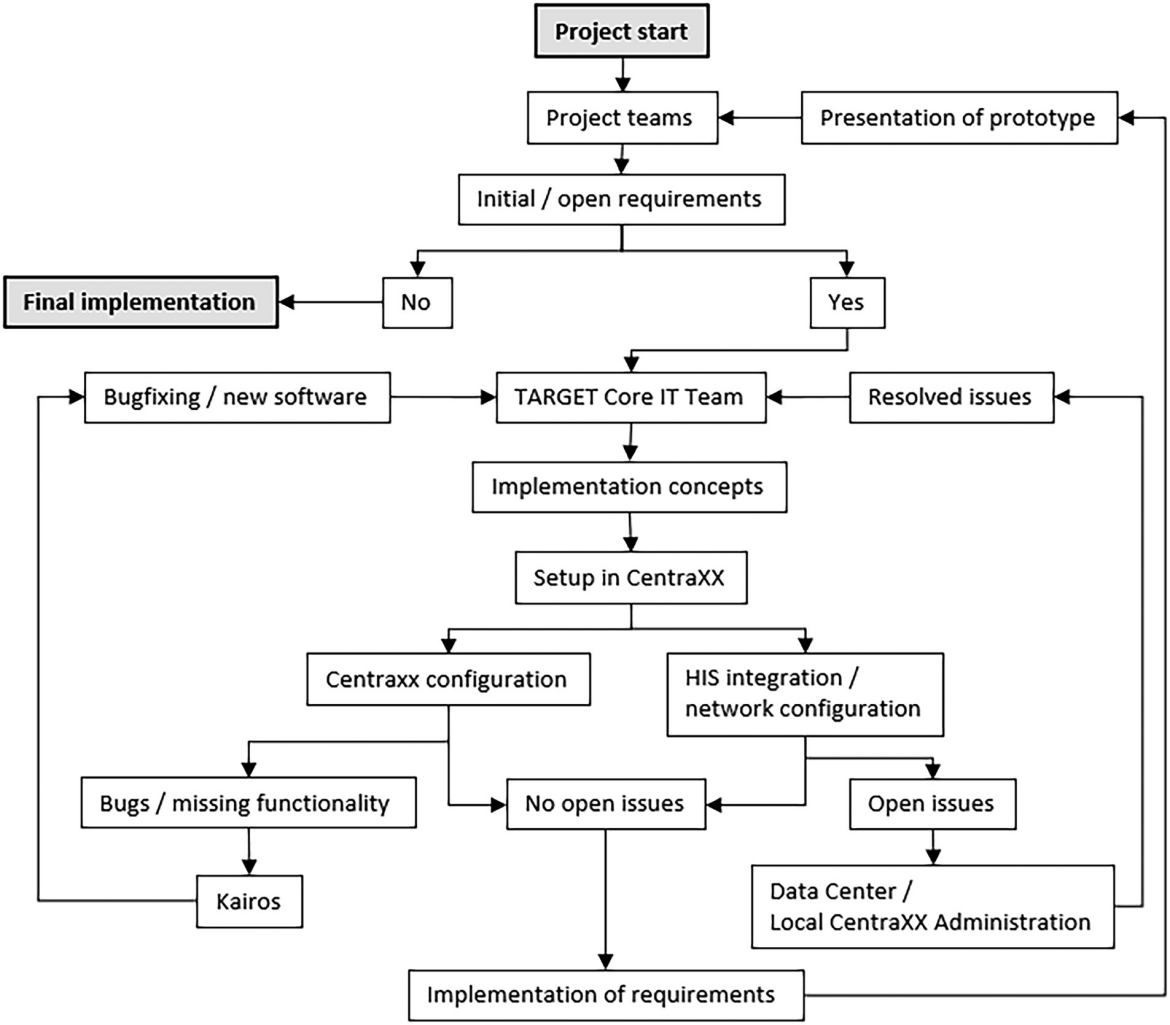
The project begins with initial requirements defined by the project teams. Persistent requirements are passed to the TARGET Core IT Team, who develop implementation concepts and CentraXX setup methods. If issues arise during CentraXX configuration, Kairos was engaged to resolve them; problems regarding HIS rollout or network configuration were coordinated with the Data Center or local CentraXX administration. Implemented requirements were demonstrated to the project teams until satisfaction was achieved.

The given requirements were ultimately implemented using the software CentraXX, a software product developed by Kairos,^13^ configured by the internal IT-coordination team. Necessary extensions to CentraXX were carried out directly by Kairos/IQVIA at the request of the internal IT-coordination team. Integration into the hospital system was performed in consultation with the hospital's local data center and the local CentraXX administration. The organizational flow during the implementation phase can be seen in Figure 1.

The implementation of the architecture at the LMU Hospital began in March 2022 and was ready for use in February 2023.

## Results

### Architecture and data privacy protection

**Figure 2.**
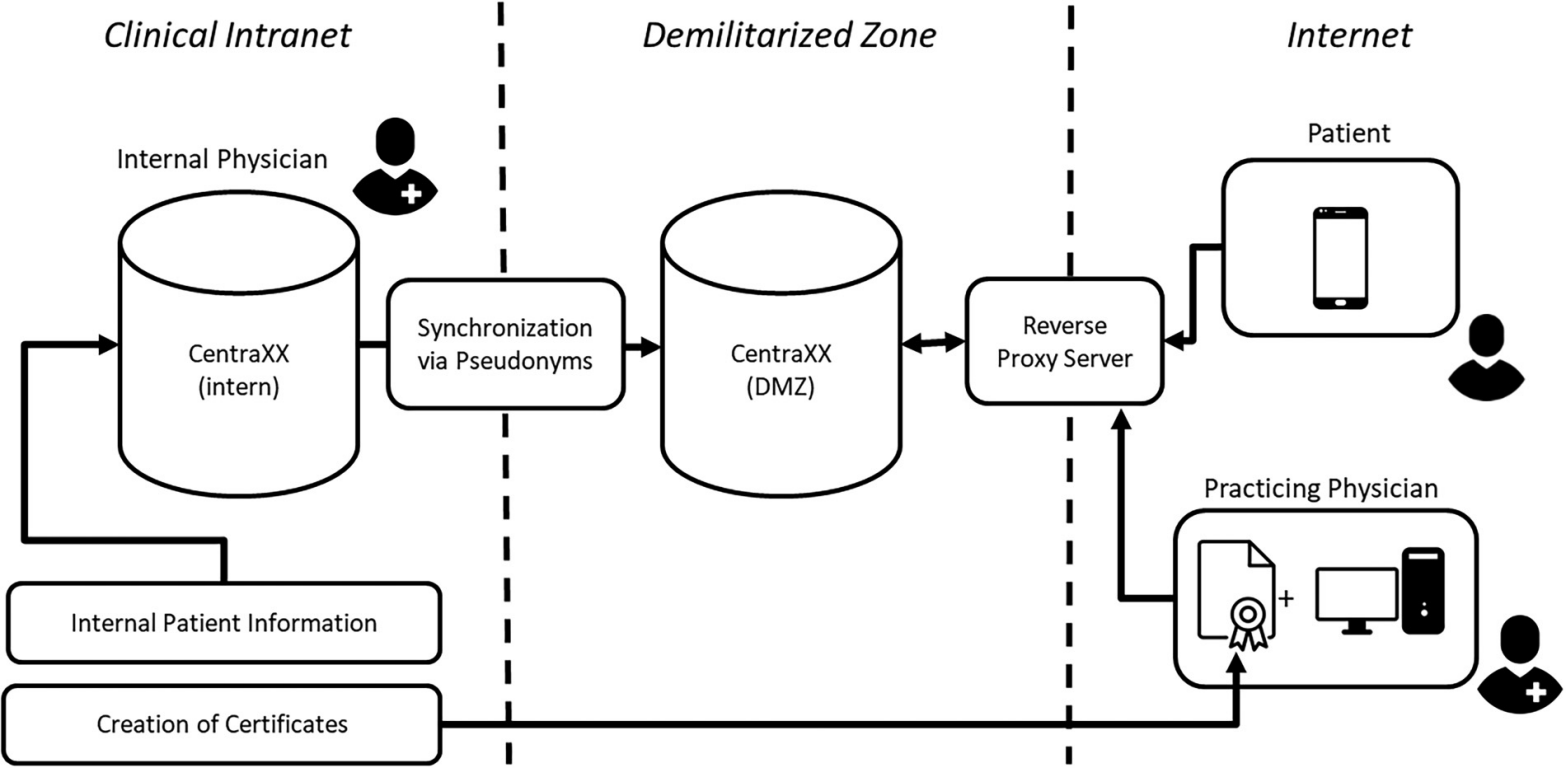
This graphic presents an overview of the initially proposed infrastructure. The internal CentraXX instance, which is linked to certain segments of the hospital's internal patient information, is synchronized with another instance located in the demilitarized zone (DMZ) through the use of pseudonyms. This secondary instance will contain all the necessary information pertinent to the TARGET trial. Internal physicians access the CentraXX instances through the hospital's intranet. On the other hand, patients and practicing physicians access the system from the internet. Patients utilize the patient app installed on their smartphones, while practicing physicians connect via a web browser and a reverse proxy that exclusively accepts connections with pre-established certificates.

CentraXX primarily emphasizes biobanking and study management and has gained significant adoption among German university hospitals (34 out of 36). Of central importance to our aim was the fact that CentraXX allows the design of individual electronic Case Report Forms (eCRFs), already possesses a patient record (**EHR**) and offers an optional oncology module (**Documentation**) that expands upon this record. The CentraXX product also includes a patient app (**Patient-access**, **Authorization**), essentially a patient-reported outcome (PRO) module that enables patients to interact with the CentraXX instance via smartphone or tablet. Figure 2 illustrates the initially planned architecture at both the site of the Ludwig-Maximilians-University (LMU) hospital as well as the Rechts der Isar (MRI) University Hospital, with both hospitals being part of the CCCM.

Even prior to the initiation of TARGET, we had two instances of CentraXX deployed at the LMU hospital. The internal instance resides within the medical network of the LMU hospital and is not accessible externally. This instance has already been integrated with patient master data, as well as information pertaining to surgeries, laboratory results, and other relevant data.

In the past, a second instance of CentraXX was established within a demilitarized zone (DMZ)^14^ as part of the COVID-associated RisCoin study.^15^ A DMZ, acting as a buffer zone between the internet and an organization's internal network, serves to protect internal networks from external threats while allowing access to selected external connections and containing only necessary and minimal data which always remains at the hospital. This adds an additional layer of security. At that time, the two CentraXX systems were not synchronized, and no synchronization was planned. It is noteworthy that multiple DMZs are currently operational within the LMU hospital.

The DMZ was made accessible to patient app users through a reverse proxy server. The reverse proxy server functions as an intermediary between the clients (users of the patient app) and the servers.

To ensure the CentraXX instance's reachability from the internet, a hostname was established via the Domain Name System (DNS) and a server certificate, authenticated by an official root certification authority (CA), was installed on both the reverse proxy and the CentraXX system.

The reverse proxy inspects and filters incoming requests and forwards them to specified URLs of the appropriate backend server. Access is restricted to Transport Layer Security (TLS) version 1.2 or higher, ensuring secure communication. Additionally, BSI-recommended cipher suites with Perfect Forward Secrecy (PFS) are employed as the preferred security configuration. The firewall between the intranet and DMZ permits access from the intranet to port 443/TCP of both the reverse proxy and the CentraXX system in the DMZ, and access is possible through the same URLs as from the internet.

Patients receive an individual QR code (supplemental material) upon recruitment into the project, which they enter into their smartphones to establish a connection via the patient app toward the DMZ.

Aside from reaching CentraXX via the patient app, an additional access method for web-browsers was implemented. For this, internal TARGET IT administrators were granted the ability to issue client certificates via a dedicated certification authority (TARGET CA) using OpenSSL.^16^ When provided to external partners and integrated into a web browser as client certificates, these certificates enable partners to connect via their desktop PCs to the CentraXX instance. The reverse proxy verifies the validity of the presented client certificate, that is, that it was issued by the TARGET CA. If valid, the reverse proxy grants access to the CentraXX user interface. Once an external partner establishes a connection using this method, their credentials are requested. Physicians have visibility only into the patients they or their clinic partners have included in the project, without access to other patients’ information. Complete collective data access is restricted to internal project personnel based on patient consent (**One-framework**, **Physician-access**). Within the TARGET team, varying levels of permissions are implemented, controlled centrally and with high granularity via CentraXX. In summary, the DMZ-CentraXX instance is accessible only with a client certificate and displays data and patient profiles to individuals based on their authorization. Data privacy is further ensured through the separation of the DMZ instance from the internal primary instance. The software components have undergone technical data protection clearance by the Privacy Protection Commissioner of LMU (**Data-protection**). Additionally, the study obtained ethical approval from the Ethics Committee of the medical faculty of the LMU hospital (project number: 22–1000).

**Figure 3.**
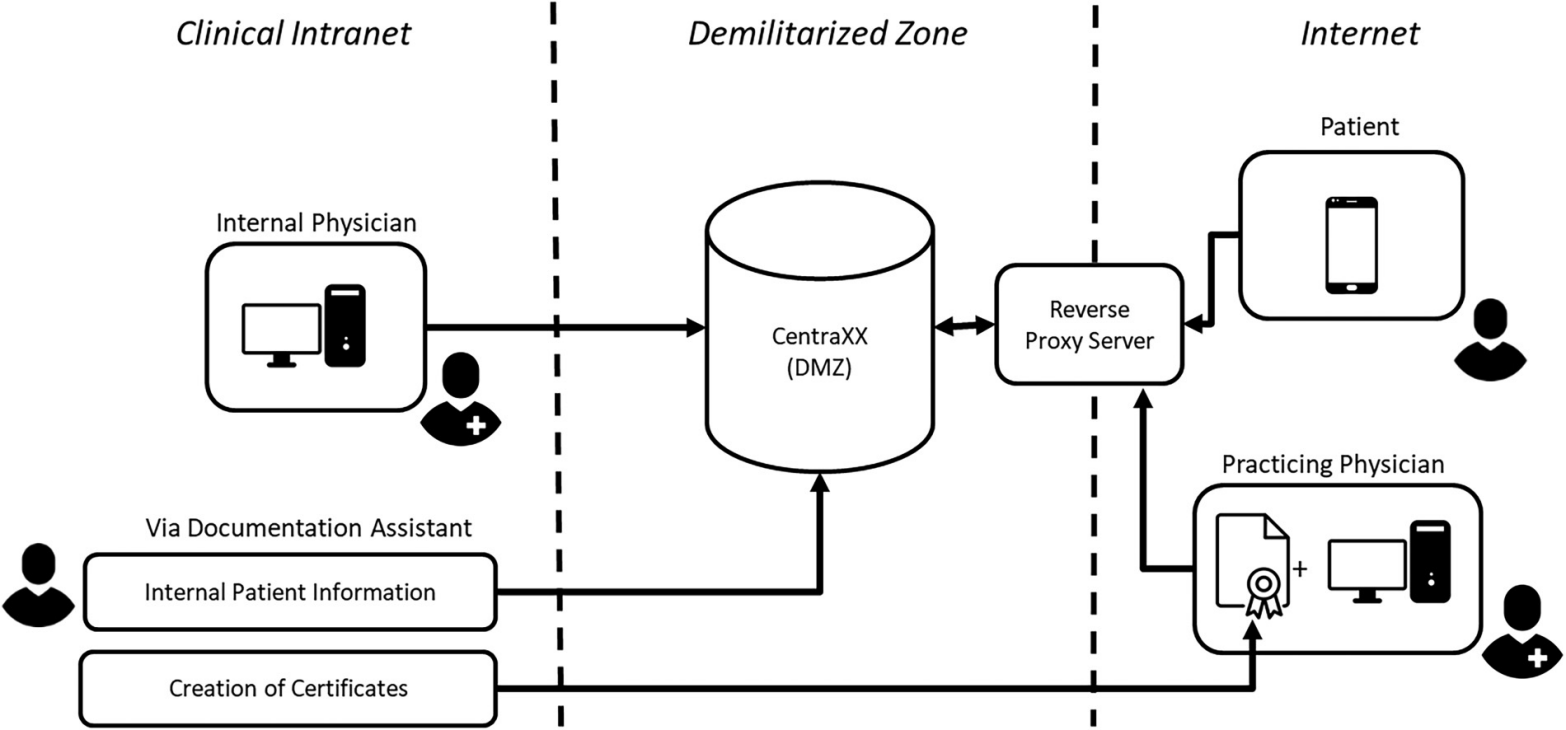
Contrary to the initially proposed architecture, internal physicians now establish a direct connection to the CentraXX system located in the demilitarized zone (DMZ). As a result, the electronic health record (EHR) in the DMZ no longer automatically synchronizes information from the internal system. Instead, data is currently entered into the DMZ EHR manually through the use of a documentation assistant.

Requirement (**Synchronization**) is currently subject to partial implementation, as shown in Figure 3 and discussed in the corresponding section of this work. While it would be technically possible to connect the primary instance to the DMZ instance, for example via insurance numbers, and to transfer information from the internal to the DMZ instance, this synchronization is currently performed manually as part of the project's documentation, sharing essential findings, etc. from inside to outside.

### EHR, workflows, eCRFs, reports, and communication

After explaining the primary architecture, attention now shifts to data contents. The EHR is already integrated into CentraXX, encompassing comprehensive patient information.

**Figure 4.**
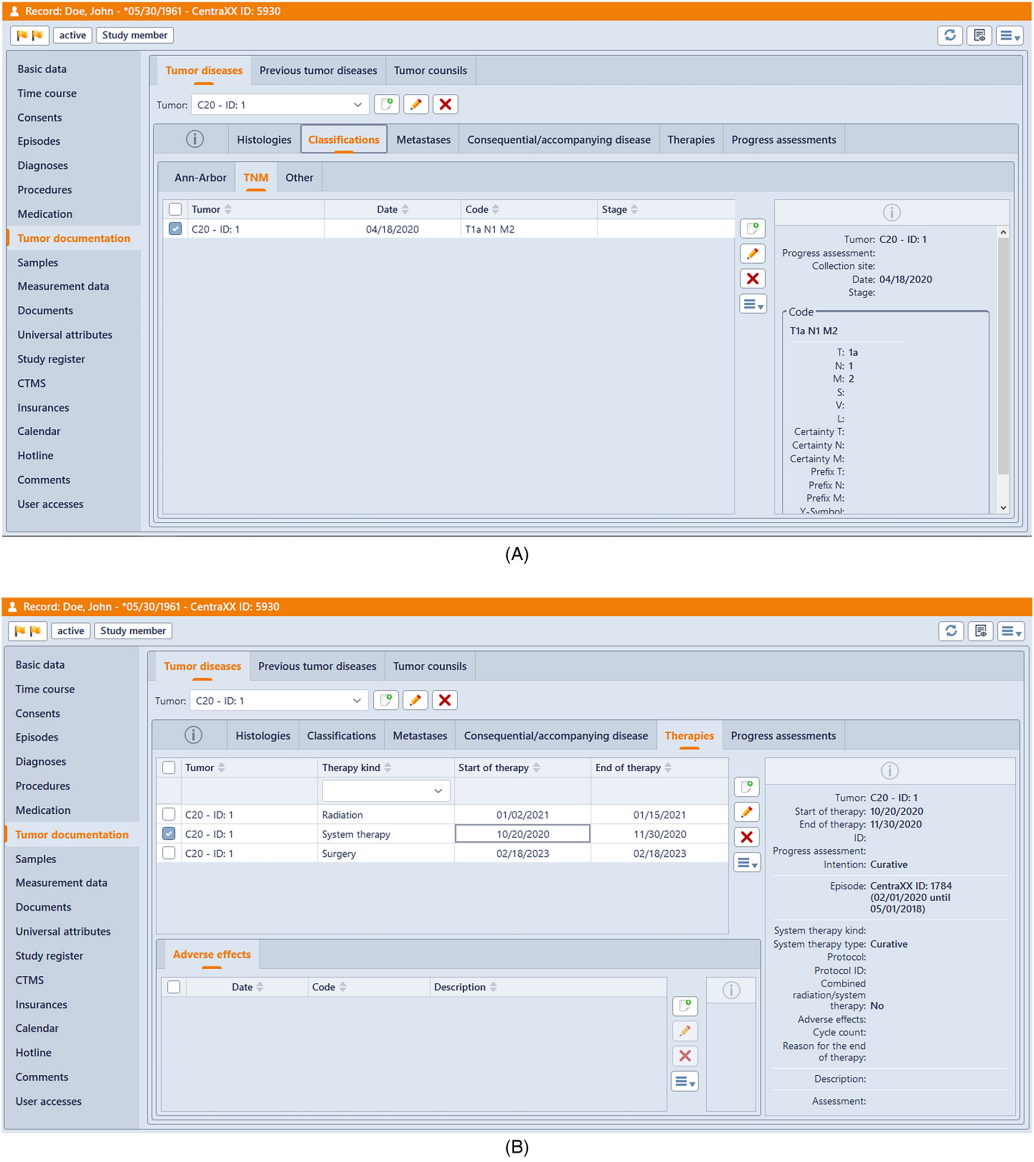
The provided screenshots depict the documentation of a simulated patient within an electronic health record (EHR) system, showcasing its integrated oncology module and a selection of its functionalities. Figure 4A illustrates the process of documenting the initial assessment, while Figure 4B showcases the recorded therapies administered to the patient.

The record has been augmented through the incorporation of the oncology module (see Figure 4), designed to facilitate the capture of oncological features.

**Figure 5.**
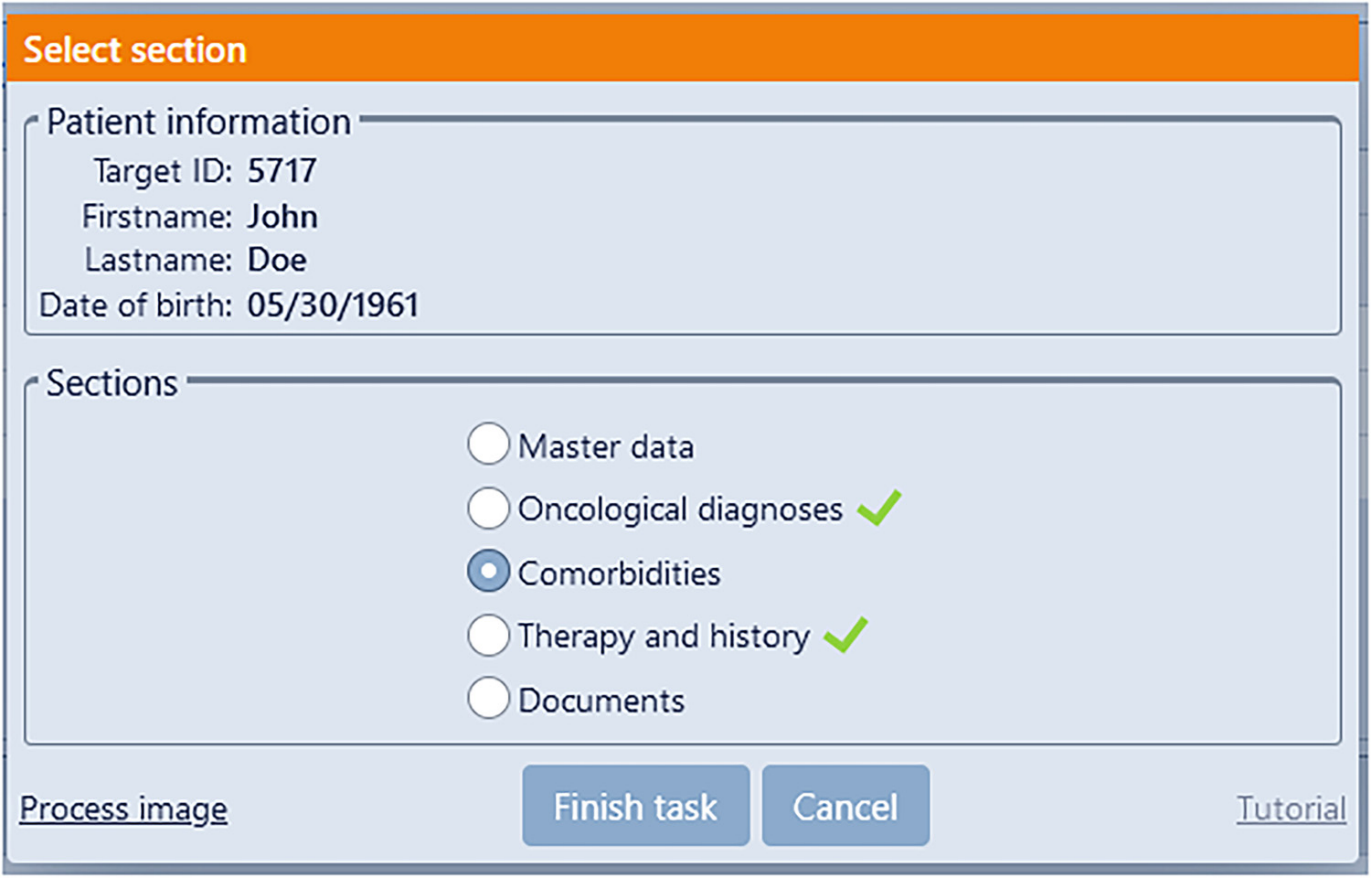
The start screen of the digital workflow process guides practitioners through the documentation of a patient within the electronic health record (EHR). It provides support to the physician in completing only the essential documentation for given categories (e.g. assessment of diagnosis), thereby facilitating an efficient documentation process.

**Figure 6.**
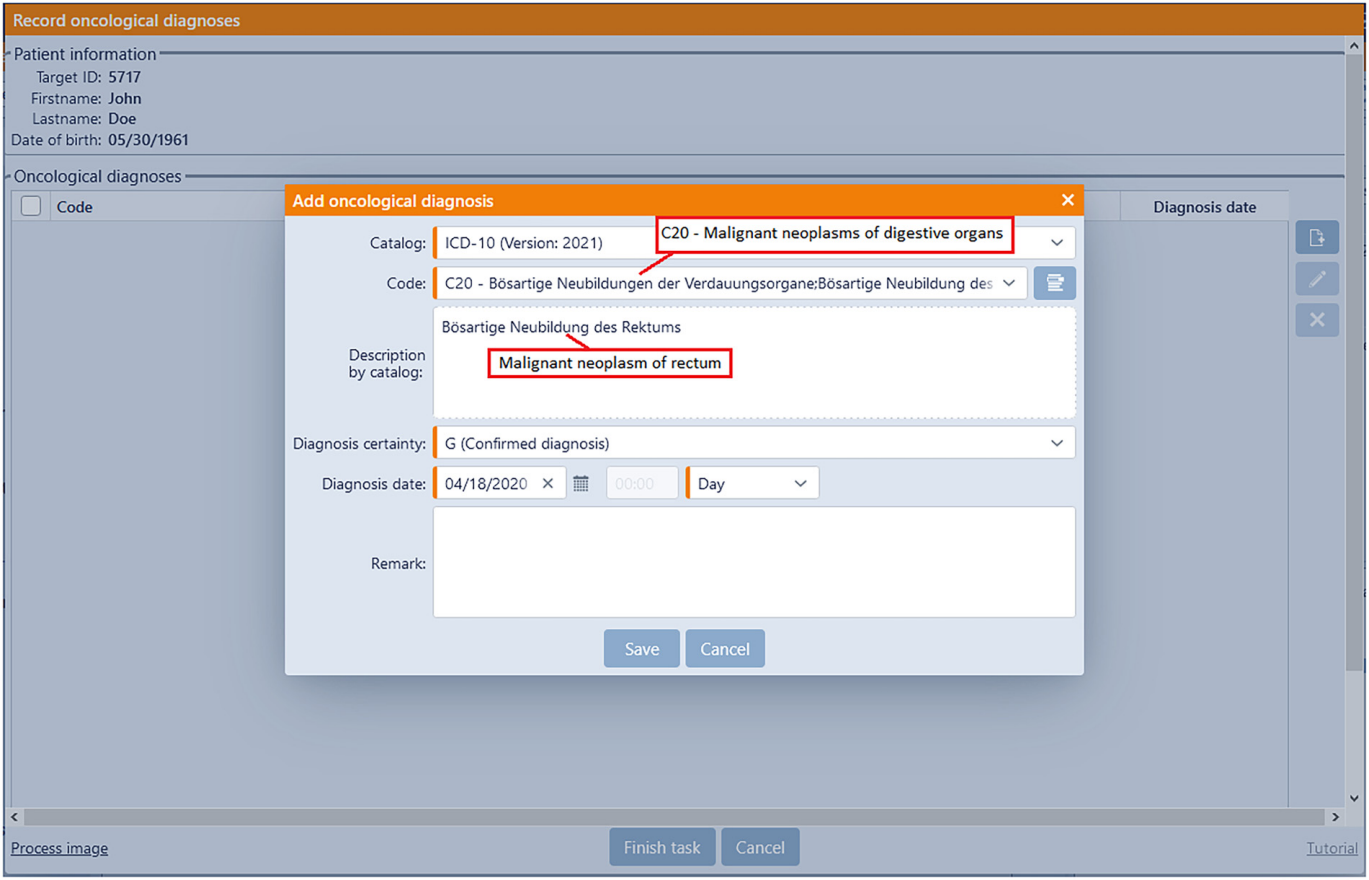
The workflow view presented here pertains to the inclusion of a diagnosis. Although the implemented CentraXX electronic health record (EHR) system potentially encompasses a wide range of data fields, the workflow solely focuses on collecting the mandatory (highlighted in orange) and optional fields necessary for the trial and transcribes them into general EHR.

However, the freedom of documentation in the EHR can pose challenges for external practitioners, who may encounter uncertainties regarding what information should be documented and where. To address this, a set of comprehensive paper-based guidelines was developed during the initial project phase, offering step-by-step instructions to primary care physicians on what and how to document within the patient record. To improve on this, a digital workflow was introduced providing dedicated input forms digitally guiding through the required documentation process, with the collected data automatically stored within the record. Figure 5 provides an overview of the high-level workflow categories, while Figure 6 gives a short impression how it guides through actual documentation.

CentraXX incorporates a workflow engine, and the specific workflow for TARGET was custom-tailored to enhance usability in accordance with (**Usability-physicians**).

Another workflow, referred to as a contact platform, enables the recruiting oncologists to establish communication with the internal TARGET team, in order to assess a patient's eligibility for the study.

**Figure 7.**
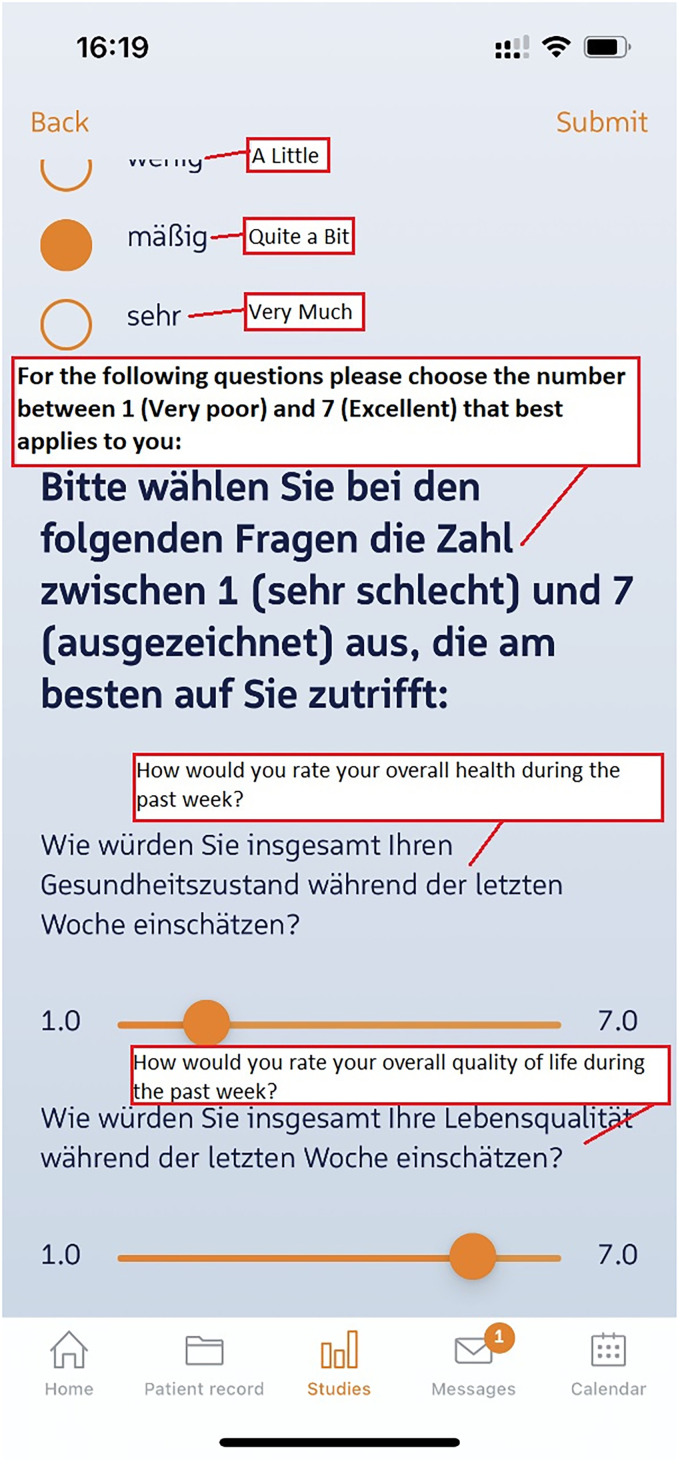
The screenshot gives an impression of the questionnaire format as perceived by the patients. This example features both multiple choice questions as well as a rating system (in this case a patient's estimation for general wellbeing and life quality in the last week).

Various questionnaires created for various topics (see Table 1) were directly implemented in CentraXX in the form of eCRFs and integrated into the patient app (see Figure 7 as an example):

**Table 1.** This overview presents details on the questionnaires and timers within the system.

Questionnaire name	Time trigger
Welcome questionnaire	Start of study
QLQ: Quality of life questionnaire (based on EORTC QLQ-C30)^17^	Start of study; after 6 months
Functions (subset of QLQ)	After 3 months
Symptoms (subset of QLQ)	Every week
Information-seeking behavior regarding transfers (FCCHL)^18^	After a month
Care coordination^19^	After a month; after 6 months
Distress thermometer^20^	Every 3 months

Initially, patients can view all questionnaires upon starting. However, the ability to edit these questionnaires is restricted until the designated time trigger is reached, which is indicated by a message within the application.

The questionnaires are time-triggered within the patient app and made available to the patient after a certain period of time.

Another extensive questionnaire on the topic of decision support for patients was implemented in LimeSurvey instead of directly within the patient app due to specific requirements.^21^ However, the access link was referenced through the patient app.

**Figure 8.**
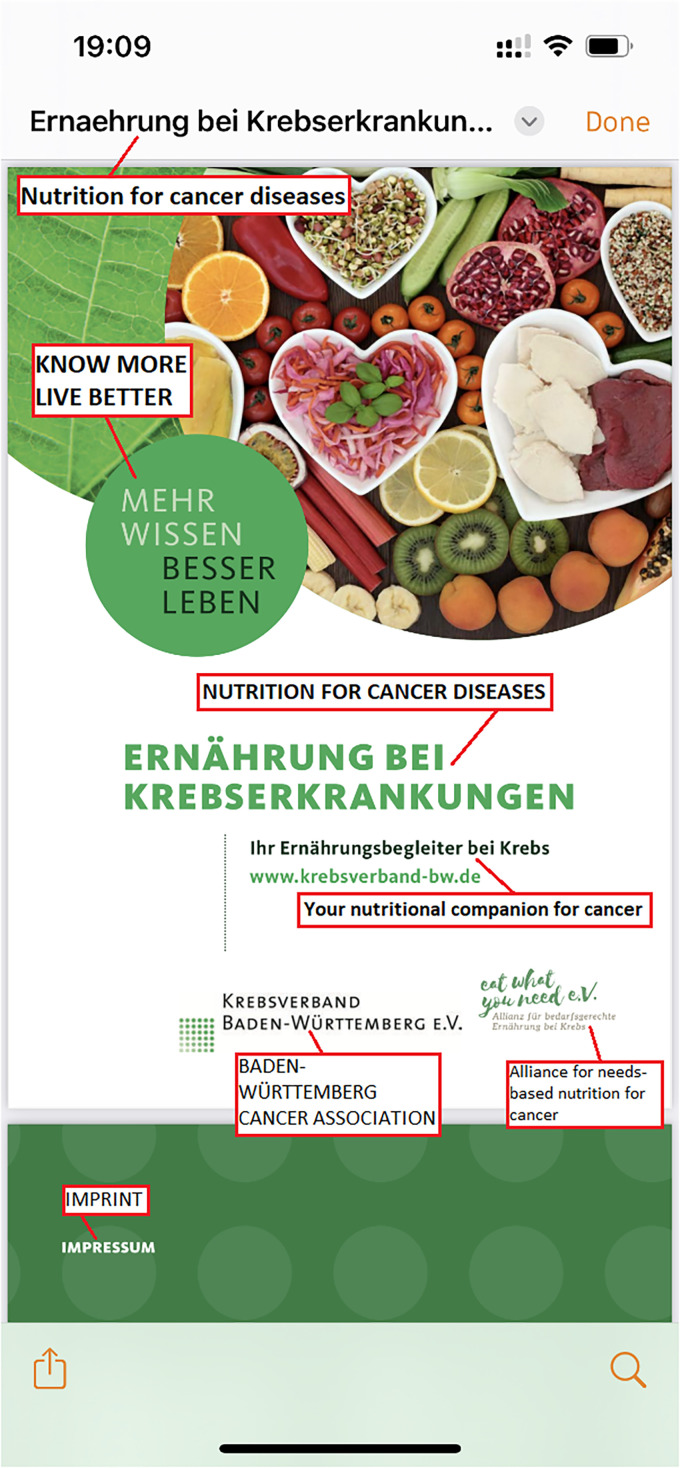
The provided example showcases the inclusion of supporting material, specifically related to nutrition (German), within the application. This material is seamlessly integrated into the app and accessible to the patient.

In addition, further informational materials and guidelines on nutrition and physical activity were provided (see Figure 8 as an example).

**Figure 9.**
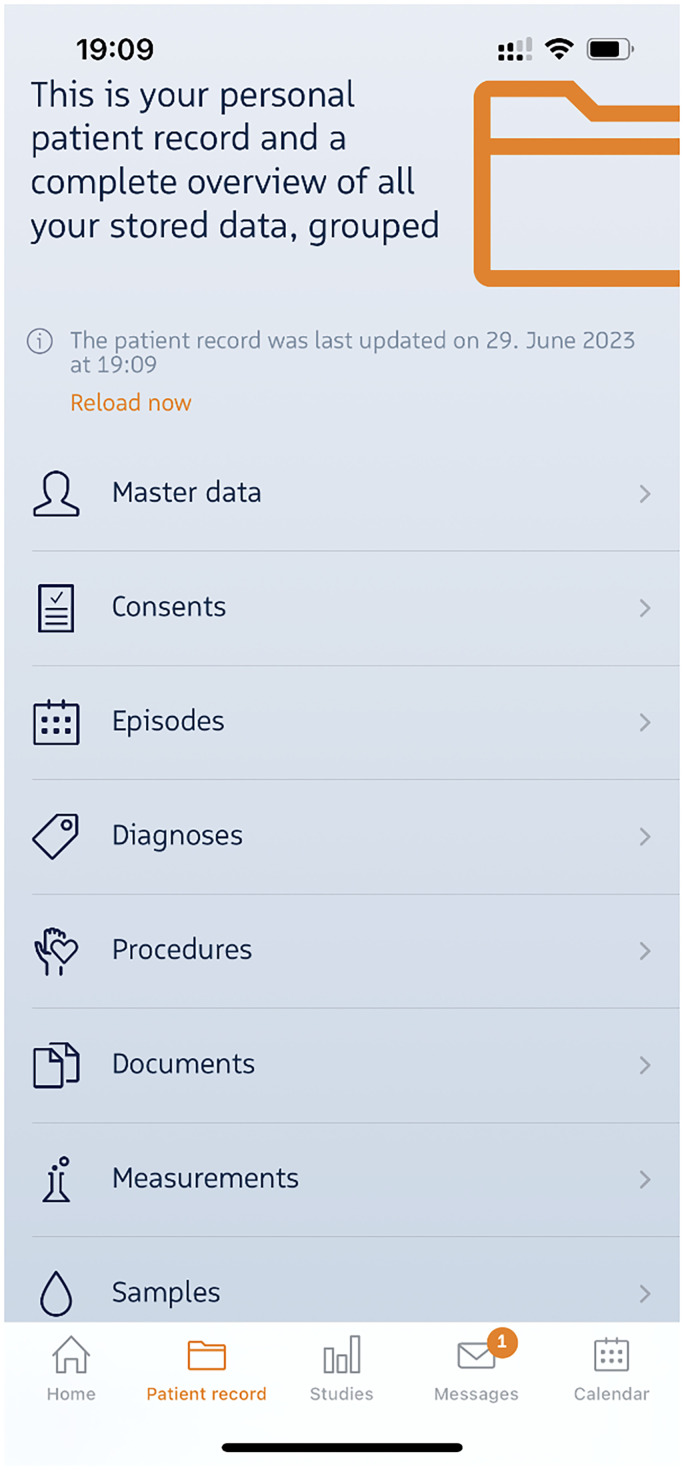
The app's general interface encompasses various features and functionalities. In the documents section, the display of medical letters is contingent upon two conditions: first, a consultation with a doctor must have taken place, and second, the necessary permissions must be granted.

Another important feature of the patient app is access to the patient's EHR, which has only been partially granted (particularly for structured documentation). Medical letters, however, are only made available to the patient after consultation with the treating physician and upon authorization in CentraXX (Figure 9).

Upon manual authorization by an Onco coach, a patient can get a chat functionality within the patient app, thereby granting the capability to contact the CCCM TARGET team (**Messaging**).

CentraXX facilitates the creation of reports, typically providing overviews that respond to events or temporal intervals. Additionally, we have integrated the CentraXX internal reports with a mail server, enabling the internal staff to receive email notifications regarding the creation of reports. However, the technical implementation of reports presented some challenges and was realized through the involvement of Kairos in the project (**Reporting**). The following reports, along with their respective recipients, have been established (refer to Table 2).

**Table 2.** This overview presents a comprehensive view of all reports, along with their respective TARGET recipient groups and the corresponding timestamps indicating when they become accessible.

Type	Time trigger	Recipient
Overview of all new distress values	Daily	Psycho-oncology team
Overview of how long patients have not filled out the welcome questionnaire	Weekly report of all patients with at least 7 days of inactivity	CCCM TARGET team
When was something changed in the EHR by the recruiting oncologist?	Twice a week	CCCM TARGET team
All structured content of a patient's EHR in the form of a medical letter	On-demand retrieval only	CCCM clinicians, Onco coach
Overview of remaining time for patients with < 30 days remaining in the study	Biweekly	CCCM TARGET team
Overview of admitted and declined patients	Weekly	CCCM TARGET team, Onco coach
Overview of unanswered patient inquiries (chat functionality)	Weekly	Onco coach

### Data export and data analysis

The TARGET project has been registered in the interoperability register.^22^ This means that all captured data can be exported in common formats such as CSV or XML. For analysis purposes, minimal datasets should be created for the respective associated analysis teams. This will be done manually by internal staff. These datasets will be pseudonymized using a custom-written tool and then sent to the project teams according to the given patient consent. The recruitment centers have also been assigned pseudonyms in this process (**Evaluation**).

The data transfer will be facilitated through the LMU Databox, a secure connection for data transmission established by the LMU hospital.

### Offered IT support services

The entire IT concept is supported by manual assistance, support, and informational materials. For TARGET, an IT responsible person has been hired and embedded in the internal CCCM IT team, which is generally independent of TARGET. This ensures continued support even in the event of the dedicated person's absence. The designated person or their representative creates certificates for the connection with CentraXX and integrates them directly into the browsers in the recruiting centers.

This visit is accompanied by comprehensive training, where recruiting doctors are instructed on how to use the EHR. In addition, doctors are also provided with an explanation of the process of equipping a patient with the patient app (QR code) and initially registering patients in TARGET.

Furthermore, a separate patient folder (paper-based) is provided to the medical centers. This folder includes center information, including a written summary of the previous training, introduction of the different TARGET project teams, as well as patient information—meaning all documents that the doctor needs to recruit the patient.

Preparations have already been made by the internal TARGET team, especially regarding the patient app. Five patients per center are initially created with a pseudonym in both CentraXX and the patient folder. During recruitment, the doctor only needs to add the real data while the patient's individual QR codes are already available. This relieves the recruiting doctors and simplifies the process.

Additionally, an IT support email has been established for the recruiting practices to directly contact the TARGET IT team in case of any problems.

Within the study folder, essential study documents for each prepared patient, as well as instructions for using the patient app, individual questionnaires, and other additional offerings, have been included.

## Discussion

The project focused on CentraXX and its integrated EHR, inspired by other projects like DataBox, ACHT, and SekTOR-HF.^10–12^ Alternatives to CentraXX could have been for instance IBM Clinical Development, secuTrialR, or tranSMART.^23–26^

The decision to use CentraXX was due to oncological parameters already being integrated through its oncology module, reducing the need for additional fields as well as, insights from the Charitee's DataBox project which also used CentraXX and thus provided guidance on connecting with outpatient physicians. The inclusion of a patient app and remote access for physicians fulfilled the key requirements.

Additionally, it should be noted that CentraXX was already in place at the LMU hospital, and the patient app had been introduced through the RISCOIN study. Thus, the tele medical connectivity to local oncologists, introduction of eCRFs, reports, and workflows, was the primary task. Despite a tight schedule, the technical implementation was completed within a year and a half.

Originally, a full rollout was planned for the partner clinic at MRI, but due to time constraints and the initial absence of a campus-wide CentraXX installation there, remote access for CCCM physicians at MRI was established similarly to that of outpatient physicians. The postponement of the internal instance synchronization was driven by the prioritization of immediate requirements and existing time constraints. The potential for the future deployment of synchronization remains viable. Implementation and synchronization at both university clinics may be pursued in the future, but the architecture and security concepts currently function as intended for both sites.

While the patient app was valuable, especially for shorter questionnaires, it was less suitable for more extensive questionnaires. Due to these shortcomings, the decision support tool, initially intended to be implemented in the app, was instead created in LimeSurvey. Other technical challenges, like missing push notifications, that arose during the project were mostly resolved.

Although the app was satisfactory, there are several comparable products in the field of PRO systems that may offer similar or superior functionality, such as for example, CANKADO.^27–29^ However, the integration of the app into the CentraXX framework was advantageous, eliminating the need for additional Extract, Transform, Load (ETL) jobs.^30^ Achieving complete flexibility in displaying eCRFs likely requires custom web programming, which entails higher effort.

In terms of organizational challenges, managing the many requirements from the various project teams and tempering their expectations were certainly difficult. The initial phase was particularly challenging, as IT typically plays a crucial role at the start of the project, yet the financial resources are distributed evenly over the project's duration. Rosa et al. provide a comprehensive overview of similar pitfalls and other organizational challenges associated with the implementation of IT in medical trials.^31^

The study aims to validate the technical usability, and while quantified answers are not available in this work yet, subjective experiences can be reported. The first oncologists accepted the concept well, and the accompanying IT training was appreciated. Technical problems were minimal with the assistance of on-site support, and the preparation of study materials, such as QR codes, facilitated physicians’ work. Surprisingly, working directly with the EHR according to the instructions did not pose too much of a problem for the physicians. Still, the workflow, which simplified data entry, provided additional relief. The technical support email was rarely used.

Physicians and staff at the CCCM had little trouble understanding the system. Reports sent via email alleviated the workload associated with logging in and kept staff members up to date.

In the early phase of the study, patient engagement with the digital offerings was observed, but the potential for questionnaire and offering overload was acknowledged. Further observation is required to assess the acceptance and potential burden on patients. Quantitative and qualitative results on usability and technical acceptance are expected to be available after the project's completion, likely in the years 2026/2027, along with the other findings. Although informed consent is generally obtained for the subjects of the TARGET study, it is not relevant for this work, which presents the IT concept and its implementation as the primary results.

We believe that the IT concept of the TARGET study could serve as a potential blueprint for similar projects in the medical domain, not limited to cancer research. Potential improvements could certainly be made in the area of interfaces. For instance, it would be conceivable to directly connect the documentation systems of medical practices to a system like CentraXX, thereby alleviating part of the documentation workload. This was an idea we pursued in the early phases of the project; however, due to the heterogeneity of the systems used by medical practices, this requirement was out of scope. Nonetheless, appropriate technical standards for data exchange could bring significant improvements in this area.

## Conclusion

In conclusion, the trial's IT implementation successfully met a significant portion of the early design requirements. The establishment of the given architecture can serve as a potential blueprint for future outreach projects. Institutions without CentraXX can find inspiration in this project to adopt comparable technical and data protection infrastructures. As the trial remains ongoing, some issues might receive further refinement and improvement.

## Supplemental Material

sj-jpg-1-dhj-10.1177_20552076241272709 - Supplemental material for Bridging the gap: Leveraging telemedicine and IT infrastructure to connect outpatient oncology practices with specialized expert teams in the management of rare tumors Supplemental material, sj-jpg-1-dhj-10.1177_20552076241272709 for Bridging the gap: Leveraging telemedicine and IT infrastructure to connect outpatient oncology practices with specialized expert teams in the management of rare tumors by Julia Kasprzak, Timothy Goering, Karin Berger-Thürmel, Vanessa Kratzer, Wuthichai Prompinit, Sven P. Wichert, Simon Leutner, Norbert Langermann, Michael von Bergwelt-Baildon, Volker Heinemann, Hana Algül, Martin Zünkeler, Daniel Nasseh and on behalf of the TARGET Group in DIGITAL HEALTH
