# Supplementary figures and images for: Bridging the gap: Leveraging telemedicine and IT infrastructure to connect outpatient oncology practices with specialized expert teams in the management of rare tumors
Source: Digit Health. 2024 Oct 9;10:20552076241272709. doi: 10.1177/20552076241272709 (PMC11468480; doi:10.1177/20552076241272709)

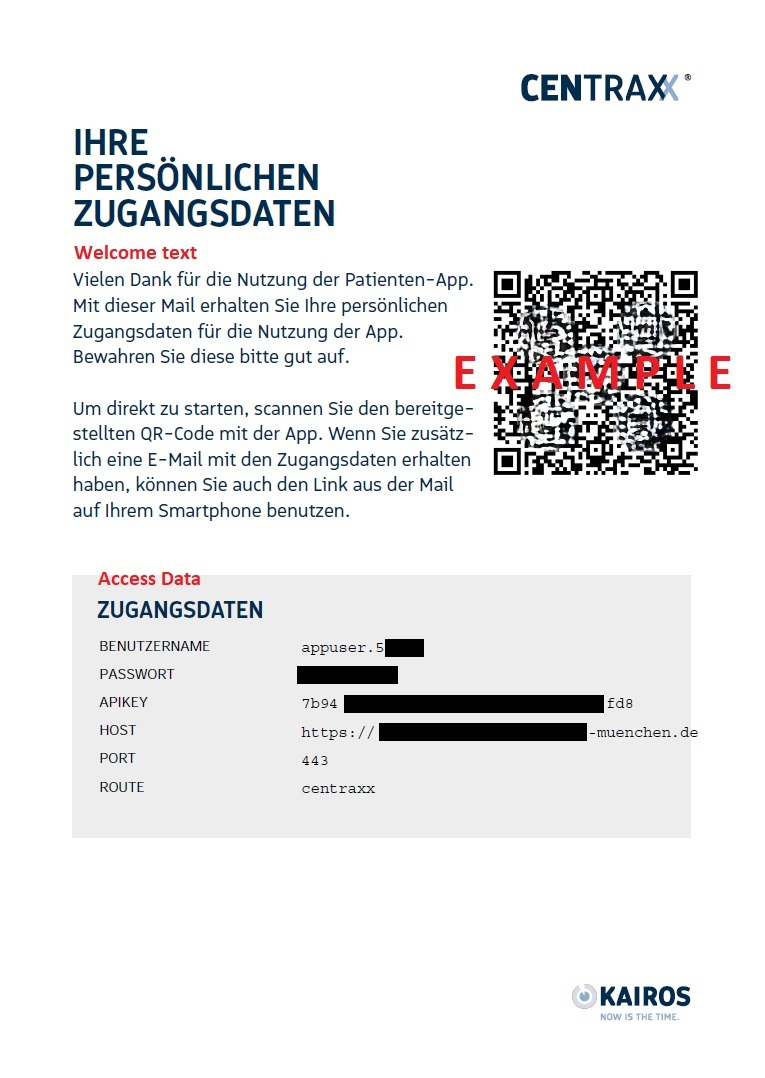

Supplement: sj-jpg-1-dhj-10.1177_20552076241272709 - Supplemental material for Bridging the gap: Leveraging telemedicine and IT infrastructure to connect outpatient oncology practices with specialized expert teams in the management of rare tumors [file sj-jpg-1-dhj-10.1177_20552076241272709.jpg]
